# Mobile eHealth solution (ePRO)

**DOI:** 10.1186/2043-9113-5-S1-S14

**Published:** 2015-05-22

**Authors:** Stanisław Saganowski, Piotr Bródka, Andrzej Misiaszek, Kazimierz Fraczkowski

**Affiliations:** 1 Wroclaw University of Technology, 50-370, Wroclaw, Poland

## Characterisation

ePRO (electronic Patient Reported Outcome), PROM (Patient Reported Outcome Measures), Android, iOS, TRANSFoRm, clinical trials.

## Description

The mobile eHealth solution developed in the TRANSFoRm project is part of the TRANSFoRm platform to support the identification of prevalent and incident cases in the GORD clinical trial use case, including the randomization of patients for on-demand or continuous consumption of PPIs [1]. This TRANSFoRm Study System (TSS) consists of five major parts:

• Study server – manages the connection between mobile and web applications and the TSS

• Study database – stores all information about studies, patients, randomization etc.

• Web application – enables filling in PROMs by patients and CROMs by GPs (investigators)

• Mobile application – enables filling in PROMs by the patients using smartphones or other devices

• Middleware - ESB which serves as a connection, authorization and security layer between TSS and the rest of the infrastructure

As part of this TRANSFoRm Study System data collection is done by web applications (eCRF) and mobile questionnaires (PROM). Patients report their data, for example data about their quality of life, by using mobile health applications from their homes. For this purpose, Android and iOS based applications communicate with the study server in order to authenticate a patient, retrieve a list of questionnaires to be filled out, and upload completed questionnaires. Communication between these components is done via secure SSL connections.

The mobile applications are capable of generating any questionnaire provided in the ODM standard format. After the patient has completed the questionnaire all data is packed into a XML file compatible with ODM and securely send to the server. No data remains on the mobile device, thereby providing additional protection of patient data. Applications offer several language versions; the proper one is selected based on the language chosen on the device. Recently the mobile eHealth solution is being evaluated by employing it in the GORD trial, a multicenter international randomized trial planning to recruit about 1000 patients with gastro-oesophageal reflux disease [1,2].

## Status of development

Development of the Transform Study System is completed. All components are in the testing phase and since December 2014 the validation phase is being conducted. The Android and iOS applications will be available free of charge on Google Play and App Store.

## Users

Patients participating in a study, General Practitioners (GP), researchers.

## Link

Project web site [http://www.transformproject.eu/]
